# Prognostic value of circulating tumor DNA in patients with colon cancer: Systematic review

**DOI:** 10.1371/journal.pone.0171991

**Published:** 2017-02-10

**Authors:** Gaowei Fan, Kuo Zhang, Xin Yang, Jiansheng Ding, Zujian Wang, Jinming Li

**Affiliations:** 1 National Center for Clinical Laboratories, Beijing Hospital, National Center of Gerontology, Beijing, China; 2 Department of Clinical Laboratory, Beijing Chaoyang Hospital, Capital Medical University, Beijing, China; 3 Department of Clinical Laboratory, Shunyi Women and Children’s Hospital of Beijing Children’s Hospital, Beijing, China; Hospital Authority, CHINA

## Abstract

The application of circulating tumor DNA(ctDNA) represents a non-invasive method for tumor detection. Its prognostic significance in patients with colorectal cancer is controversial. We performed a systematic review of data from published studies to assess the prognostic values of ctDNA in patients with colorectal cancer. We searched Medline, Embase, Web of Science, the Cochrane Library, and Scopus databases to identify eligible studies reporting disease-free survival (DFS) and overall survival (OS) stratified by ctDNA prior to December 6, 2016. We evaluated the quality and design of these studies. A total of 22 studies were eligible for systematic review. Among them, 11 studies investigated the prognostic value of ctDNA on disease-free survival (DFS). Seven of 11 studies showed that ctDNA was an independent variable to estimate the probability of DFS by multivariate analyses. Thirteen studies assessed the relationship between ctDNA and overall survival (OS). Eight of 13 studies showed that ctDNA was an independent predictor of worse OS through the use of multivariate analyses. This analysis provides evidence that ctDNA may be a prognostic biomarker, negatively correlated with the survival of patients with colorectal cancer.

## Introduction

Circulating free DNA with tumor-specific alterations (ctDNA) is found in serum or plasma and represents a small fraction of the total circulating free DNA. It is believed that ctDNA is shed into the bloodstream from tumor cells through apoptosis, necrosis, autophagy, necroptosis, and other physiological processes [1]. CtDNA strands are small fragments (approximately 180–200 base pairs in length), containing tumor-specific alterations in tumor suppressor genes or oncogenes, microsatellite instability, and DNA hypermethylation [2,3]. Some specific genetic alterations detected in ctDNA are driver alterations that are responsible for the initiation and progression of human cancers. Those alterations play broad roles in vivo, such as affecting genomic surveillance mechanisms and reducing cells’ ability to detect and/or repair DNA damage, which increases susceptibility to DNA damage by exogenous and endogenous carcinogens [4]. Epigenetic alterations, such as methylation of CpG islands in promoter regions, are responsible for the silencing of multiple tumor suppressor genes [4]. In some instances, hypermethylation can lead to microsatellite instability.

Virtually every type of cancer harbors genetic/epigenetic alterations. Some studies illustrated that alterations in ctDNA were in concordance with the genomic spectrum of the tumor, providing evidence that ctDNA may be a potential surrogate for the entire tumor genome. Recently, ctDNA has emerged as a non-invasive blood biomarker in tumor precision medicine. CtDNA correlates with tumor stage, tumor burden, and therapy in patients with colorectal cancer (CRC). Patients with early-stage or minimal residual disease usually have lower levels of ctDNA, making it difficult to precisely detect specific alterations. The results of intensive efforts are now evident with the development of new highly sensitive technological methods that can overcome this problem.

Comprehensive analysis of ctDNA is becoming increasingly popular. Potential applications include, but are not limited to, early detection, observation of dynamic tumor changes, assessment of tumor heterogeneity, identification of genetic/epigenetic alterations for targeted therapy, and assessment of drug resistance development [3,5].

Among the numerous possible applications, the prognostic and predictive values of ctDNA in CRC have generated the most intense interest. Studies uncovered that ctDNA could be a reliable prognostic factor correlated with poorer outcome [6]. Positive detection of ctDNA implies a high risk of recurrence or short overall survival (OS) in patients with CRC treated with surgery, chemotherapy, radiotherapy, or targeted therapy [7–9]. However, other studies found no difference in survival between ctDNA-positive and ctDNA-negative CRC [10].

To clarify the prognostic role of ctDNA in CRC, we initiated a systematic literature review to gain a better understanding of its prognostic value in patients with CRC.

## Methods

### Criteria for inclusion

Eligible studies met the following criteria: inclusion of only patients with CRC; analysis of the correlation between patient survival and ctDNA status; and inclusion of follow-up data for OS, disease-free survival (DFS), and/or cancer-specific mortality. Both prospective and retrospective cohort studies were included. Reviews, comments, and case reports were excluded.

### Search methods for identification of studies

We adhered to the Meta-analysis of Observational Studies in Epidemiology guidelines to identify eligible studies. We conducted systematic electronic searches of the Medline, Embase, Web of Science, the Cochrane Library, and Scopus databases to identify eligible studies performed prior to December 6, 2016 (no start date limit was applied). We used combinations of the following search terms: “Colonic Neoplasm,” “Neoplasm, Colonic,” “Neoplasms, Colonic,” “Colon Neoplasms,” “Colon Neoplasm,” “Neoplasm, Colon,” “Neoplasms, Colon,” “Cancer of Colon,” “Colon Cancers,” “Cancer of the Colon,” “Colonic Cancer,” “Cancer, Colonic,” “Cancers, Colonic,” “Colonic Cancers,” “Colon Cancer,” “Cancer, Colon,” “Cancers, Colon,” “colonic,” “colorectal disease,” “rectal neoplasms,” “colorectal polyps,” “sigmoid neoplasms,” “colorectal adenoma,” “circulating tumor DNA,” “ctDNA,” “cell free DNA,” “serum DNA,” “plasma DNA,” “circulating DNA,” “free DNA,” “prognosis,” “survival,” “prognostic,” and “predictive.” No restrictions were placed on the search, and relevant MeSH (Medline) or Emtree (Embase) terms were used where possible. The reference lists of relevant studies were manually searched to identify new studies. In additional to full publications, conference posters and letters that fulfilled the inclusion criteria were documented to capture grey literature. Publications written in languages other than English were also included if sufficient information was available in the abstract.

Each study was independently assessed for inclusion at least by two investigators (Gaowei Fan, Xin Yang), and discrepancies were resolved by discussion. Whenever multiple versions of reports were presented (e.g., same authors, overlapping period of study, same protocol ID), we retained the report with the largest patient population. Duplicates were removed.

### Data extraction

The following data were retrieved from the included studies: author, publication year, country in which the study was conducted, publication type, number of patients included in the analysis, percentage of male patients, tumor stage, median patient age, ctDNA panel, detection method, number of patients with ctDNA positivity, treatment, follow-up, sampling time, and outcome (DFS, OS, cancer-specific mortality). Individual investigators of the included studies were also contracted by email if essential information relevant to this systematic review was absent. Data extraction was performed by three independent investigators with a predefined information sheet (Gaowei Fan, Kuo Zhang, Xin Yang, Jiansheng Ding). Any discrepancies were resolved by discussion.

During the entire selection process, none of the authors was blinded to the source of the publications, the authors, or any other details.

### Quality assessment

We evaluated the quality of the included studies using the Cochrane Collaboration’s tool for assessing the risk of bias [11]. Specifically, studies were judged on the following criteria: (1) selection bias, defined as a clear description of the inclusion and exclusion criteria; (2) accuracy of measurements, also called measurement bias, defined as an explicit description of the ctDNA detection method; (3) exposure bias, defined as an explicit description of genetic/epigenetic alterations; (4) bias caused by incomplete follow-up, defined as a satisfactory report of the median follow-up length, follow-up range, and loss to follow-up rates, and (5) confounding bias, which included known or commonly discussed confounders in the relationship between ctDNA and survival, such as age, disease status, or other factors for which adjustment was performed.

### Measurement of treatment effect

The primary outcome was DFS. The secondary outcome was OS. For the purpose of these analyses, DFS was defined as the time from the initial treatment to the first documentation of relapse/recurrence. OS was defined as the time from the initial treatment to death.

### Ethics statement

This study was a literature-based study, and no ethics approval was needed.

## Results

### Included studies

**Fig 1:**
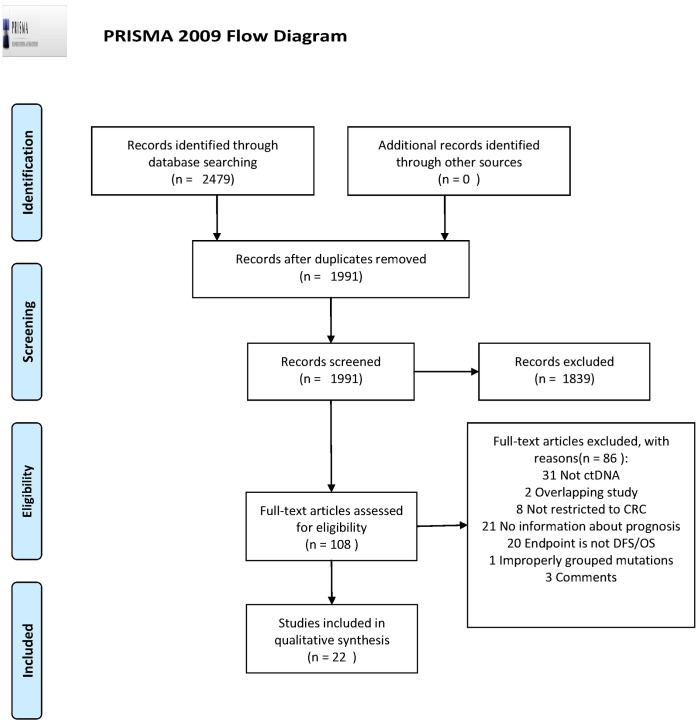
PRISMA 2009 flow diagram. PRISMA flow diagram for study selection. *From*: Moher D, Liberati A, Tetzlaff J, Altman DG, The PRISMA Group (2009). *P*referred *R*eporting *I*tems for *S*ystematic Reviews and *M*eta-*A*nalyses: The PRISMA Statement. PLoS Med 6(6): e1000097. doi:10.1371/journal.pmed1000097. **For more information, visit** www.prisma-statement.org.

A total of 2479 potential studies were initially searched. Following eligibility screening by title and abstract, 2371 studies were removed. The main reasons for exclusion were duplicative studies, reviews, non-human studies, no relevance to ctDNA, and incorrect tumor type. Of the remaining 108 studies, the full text was screened, and 86 studies were excluded because of an absence of prognosis information, not ctDNA, overlapping studies, comments, improperly grouped mutations, not DFS/OS, or the inclusion of patients with diseases other than CRC. The reasons for exclusion were listed in S1 Table. Finally, 22 studies met the inclusion criteria, and were included for descriptive summarization (Fig 1).

### Study characteristics

The included studies, published between 2002 and 2016, analyzed the relationship between ctDNA status and survival outcomes in a total of 2541 patients. The number of patients in each study ranged from 15 to 353. Eighteen studies were prospective studies, and 4 studies were retrospective studies. Of these, 21 studies were published as full publications, and the other one was conference posters.

Most studies were published in the English language. One study was published in a language other than English (with an English abstract). Patients with primary or metastatic CRC with a TNM stage of I-IV or Dukes’ stage of A, B, C, D who received surgery, chemotherapy, radiotherapy, or targeted therapy were included. The study characteristics of the patients enrolled in these studies are summarized in Table 1 and S2 Table.

**Table 1:** General characteristics of the study populations.

First author name (year)	Country	Publication type	Study design	Patients included, n	ctDNA-positive patients, n	Male (%)	Tumor stage	Median age	ctDNA panel	Detection methods	Study treatment	Sampling time	Median follow-up	Outcome
Lecomte (2002)	France	FP	PRO	37	26	NR	TNM I-IV	NR	*KRAS2*, *p16*	MASA, MSP	Sur, chem, rad	Pre-tr	22 months	OS
Spindler (2013)	Denmark	FP	PRO	97	30^KRAS^,8^BRAF^	65.6%	metastasis	66	*KRAS2*, *Braf*	ARMS-qPCR	Irinotecan, mono	Pre-tr,	NR	OS
Trevisiol (2006)	Italy	FP	PRO	15	7	NR	Dukes’ A, B, C, D	NR	*KRAS2*	ME-PCR	Sur	Pre-tr	41 months	OS
Ryan (2003)	The Netherlands	FP	PRO	85	16	NR	Dukes’ A, B, C	NR	*KRAS2*	SN-PCR, DS	Sur	Post-tr	3 years	DFS
Bai (2013)	China	FP	Retro	106	17^low mutation^, 16^high mutation^	56.6%	metastasis	55.5	*KRAS*	PNA-PCR, nested PCR	Sur, chem, cetuximab	NR	21.3 months	OS
Messaoudi (2016)	France	FP	PRO	97	38^KRAS^, 5^BRAF^	59.8%	metastasis	66.6	*KRAS*, *BRAF*	Intplex	Chemo, rad	Pre-tr	36 months	OS
Sefrioui (2015)	France	FP	PRO	16	11	37.5%	metastasis	NR	*KRAS2*	chip-based digital PCR	Chem	NR	NR	OS
Tie (2014)	America	Meeting	PRO	78	6	NR	TNM II	66	*TP53*, *APC*, *KRAS*, *NRAS*, *BRAF*, *PIK3CA*, *CTNNB1*, *SMAD4*, *FBXW7*	Safe-SeqS	Sur, chem	NR	2 years	RFS
Tie (2016)	Australia	FP	PRO	230	20	57%	TNM II	65	*TP53*, *APC*, *KRAS*	Safe-SeqS	Sur with chem or not	Post-tr	27 months	RFS
Bazan (2006)	Italy	FP	PRO	50	8^KRAS^, 8^TP 53^	NR	Primary	NR	*TP53 KRAS p16INK4A*	SSCP-PCR, MSP	Sur	NR	26 months	DFS, OS
Lin (2014)	Taiwan	FP	PRO	133	41	41.3%	TNM I-IV	NR	74 genes	MassArray	Sur	Pre-tr	62 months	OS
Lindforss (2005)	Sweden	FP	PRO	25	9	36%	TNM I-III	72	KRAS	TGGE	Sur	Pre-tr &Pro-tr	38 months	DFS
Wang (2004)	Taiwan	FP	PRO	104	36	48.7%	Dukes’ A, B, C, D	DD62.1; DU65.9	36	PCR-SSCP	Sur	Pre-tr	20 months	DFS
Herbs t(2009)	Germany	FP	Retro	106	13	NR	UICC I-III	66	*HLTF*, *HPP1*, *TPEF*	MethyLight	Sur	Pre-tr	5 years	RFS
Lee (2013)	Korea	FP	PRO	101	37	32.7%	TNM I-IV	NR	*SEPT9*	Real-time PCR	Sur, chem, rad	Pre-tr	518 days	DFS
Leung (2005)	Hong Kong	FP	PRO	49	28	36.7%	TNM I-IV	57	*APC*, *hMLH1*, *HLTF*	MethyLight	Any treat	Pre-tr	13.6 months	OS
Philipp (2012)	Germany	FP	PRO	311	48^HLTF^; 64^HPP1^	55%	TNM IV	NR	*HLTF*, *HPP1*	MSP	NR	Pre-tr,	8 years	OS
Tham (2014)	Singapore	FP	Retro	150	NR	56.7%	TNM I-II	NR	*TAC1*, *SEPT9*, *NELL1*	MSP	Sur	Pre-tr,	59 months	DFS
Wallner (2006)	Germany	FP	Retro	77	20	NR	TNM I-III	NR	*HPP1*, *HLTF*	MethyLight	NR	Pre-tr	5 years	DFS
Matthaios (2016)	Greece	FP	PRO	155	22^RASSF1A^, 29^APC^	57.4%	Dukes’ A,B,C,D	70	*RASSF1A*, *APC*	MSP	Sur	Pre-tr	NR	OS
Liu (2016)	Singapore	FP	PRO	165	82	55.2%	TNM I-IV	67	*SST*	MSP	Sur	Pre-tr	56 months	DFS, OS
Lin (2016)	Taipei	FP	PRO	353	129	60.06%	TNM I-IV	67	*AGBL4*, *FLI1*, *TWIST1*	Sequenom MassCLEAVE and MALDI-TOF	Sur	Pre-tr	56 months	DFS

PRO, prospective study; retro, retrospective study; FP, full publication article; pt, patient; NR, no report; chem, chemotherapy; FU, follow-up; pre-tr, pretreatment; sur, surgery; rad, radiotherapy; mono, monotherapy; meeting, ASCO meeting abstract; post-tr, after treatment; DS, direct sequencing; PCR, polymerase chain reaction; MASA, mutant allele-specific amplification; MSP, methylation-specific PCR; ARMS-qPCR, allele refractory mutation systems-based quantitative PCR; ME-PCR, mutant-enriched PCR; SN-PCR, semi-nested PCR; SSCP-PCR, single-strand conformation polymorphism-PCR; RFS, recurrence-free survival; OS, overall survival; DFS, disease-free survival; SSCP-PCR, single-strand conformation polymorphism-PCR; MALDI-TOF, matrix-assisted laser desorption ionization—time of flight mass spectrometry; TGGE, temperature gradient gel electrophoresis; DD, circulating DNA-detectable; DU, circulating DNA-undetectable.

**Fig 2:**
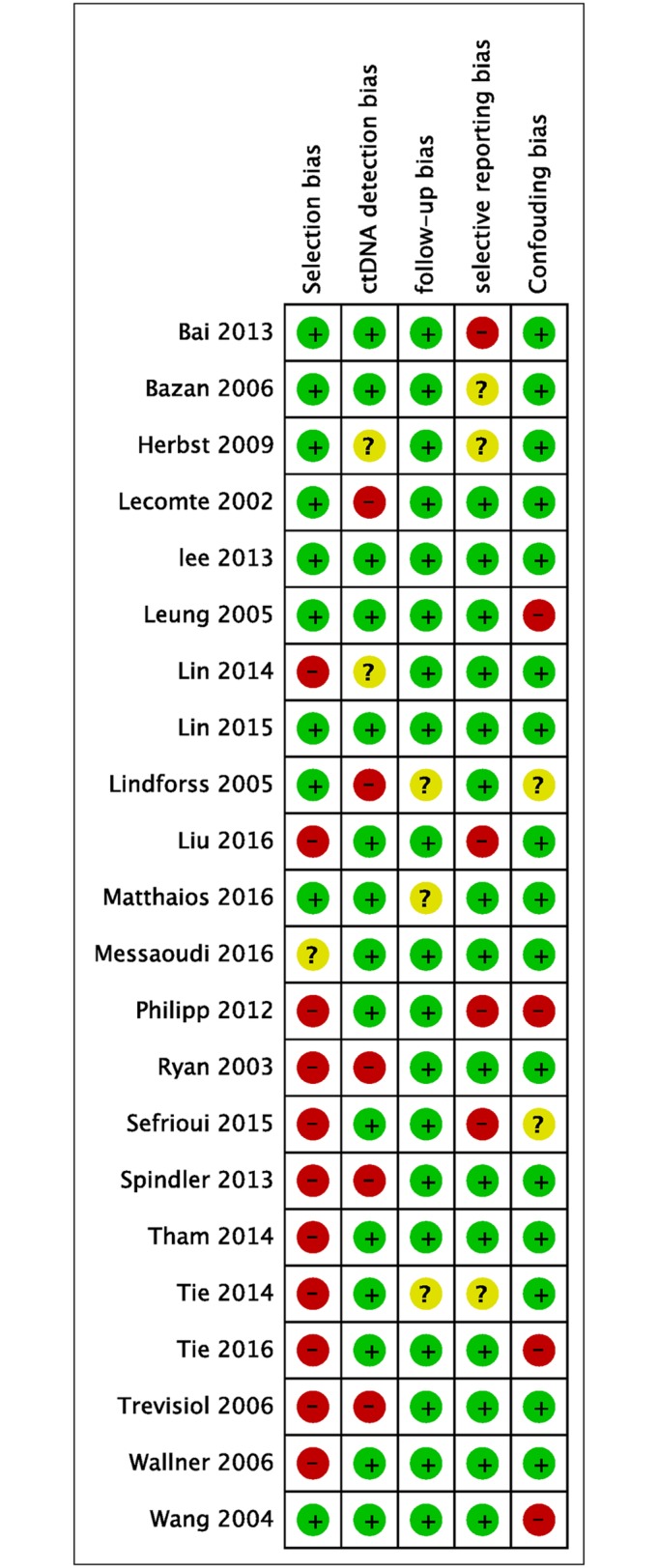
The risk of bias evaluation of the included studies. Red circles represent studies with a high risk of bias, green circles represent studies with a low risk of bias, and yellow circles represent studies with an uncertain risk of bias.

We assessed risk of bias using the Cochrane Collaboration’s tool for assessing the risk of bias in randomized trials. We categorized bias according to five domains: selection bias, ctDNA detection bias, follow-up bias, selective reporting bias, and confounding bias. We expressed risk of bias as “low risk,” “high risk,” or “unclear risk.” In most studies, inclusion and exclusion criteria for patient selection were clearly defined. These studies were rated as having a “low risk” of selection bias. Most full publication studies described the ctDNA detection method and the time to blood sample explicitly, and we rated these studies as having a “low risk” of ctDNA detection bias. Studies that reported the median follow-up length and range and the loss-to-follow-up rate clearly were rated as “low risk.” Other known factors such as tumor stage, patient age, and lymph node involvement have an effect on patient prognosis. If these factors were considered and adjusted, we rated these studies as “low risk.” The risk of bias in each included study is summarized in Fig 2, and each risk of bias feature, presented as a percentage across all included studies, can be found in S1 Fig.

### Heterogeneity in ctDNA definition

In our included studies, ctDNA status was classified in a dichotomized manner (ctDNA-positive vs. ctDNA-negative or low-level vs. high-level), with the exception of one study that classified ctDNA status in a trichotomized manner (wild-type group, low-mutation group, and high-mutation group) [12]. Some studies used both tissue and serum/plasma to detect alterations, and patients with the same detectable alterations in both tissue and serum were defined as ctDNA-positive, whereas patients with detectable tissue alterations but undetectable serum alterations were defined as ctDNA-negative [8,13–15]. Other studies defined ctDNA-positive and ctDNA-negative as detectable and undetectable serum alterations, respectively. In one study, two consecutive serum samples from the same patient had to test positive to assign a ctDNA-positive status [7].

The ctDNA panel, detection method, and time of blood sample collection also varied significantly across the included studies.

### CtDNA detection panel

The detection panels contained both genetic and epigenetic alterations. Some studies contained only genetic mutations in their detection panel, some devised a detection panel with epigenetic alterations only, and a few contained both genetic and epigenetic alterations in their detection panels.

The most commonly detected genetic mutations were *KRAS* mutations. In total, 6 of 22 studies analyzed *KRAS* only. Other mutations such as *BRAF*, *RAS*, *TP53* and *APC* mutations were often observed, usually in combination with *KRAS* mutations. For epigenetic alterations, the most commonly investigated genes were *HLTF* and *HPP1*. Other panels, such as those used by Wallner et al. (*HPP1*, *HLTF*, and *hMLH1*), Leung et al. (*APC*, *hMLH1*, and *HLTF*), Tham et al. (*TAC1*, *SEPT9*, or *NELL1*), Lin et al. (*AGBL4*, *FLI1*, *TWIST1*), Liu et al. (*SST*) and Mattaios et al. (*APC*, *RASSF1A*)were also included [16–21].

### Detection methods

The following methods were used to detect genetic mutations: single-strand conformation polymorphism-PCR, mutant allele-specific amplification, in-house assay based on allele refractory mutation systems-based quantitative PCR, mutant-enriched PCR, semi-nested enrichment technology, direct sequencing, peptide nucleic acid clamp PCR, nested primer PCR, Intplex, personalized Safe-SeqS assays and chip-based digital PCR. To detect epigenetic alterations, the most commonly used method was methylation-specific PCR and MethyLight. Besides, Sequenom MassCLEAVE base-specific cleavage method and matrix-assisted laser desorption ionization—time of flight (MALDI-TOF) mass spectrometry were also used. In some studies, ctDNA could only be detected in patients who harbored tissue alterations. Using highly sensitive technology, some studies concluded that the serum ctDNA status was in high concordance with that of the tissue.

### Sample type

Plasma or serum was used to detect ctDNA. Ten studies preferred plasma [8,9,12–14,19,22–25], whereas seven studies used serum [15,17,18,20,21,26,27,28]. The other studies did not report the sample type [6,16,29–31].

### Sample timing

Most studies obtained blood samples for ctDNA detection prior to treatment [6,8,19–21,24,26,27]. Three study collected blood samples from untreated patients before treatment or from previously treated patients at least 1 month after treatment [14,15,28]. Four studies continuously obtained samples both prior to treatment and at each visit during the follow-up period [9,16,18,25]. Three studies chose to obtain postoperative blood samples and follow-up samples [7,13,29]. The rest of the studies did not report the time at which the blood samples were collected.

### Treatment

Treatment varied across studies. The different treatments included surgery, chemotherapy, targeted therapy, and radiotherapy as well as a number of other therapeutic intervention options.

### Relationship between ctDNA and DFS

Eleven studies provided information regarding the association between ctDNA status and DFS [7,13,16,19,20,23,25,27,28,32,33]. Six of them calculated DFS according to the Kaplan-Meier method. Eight of 11 studies performed multivariable analyses to evaluate the independent prognostic effects of ctDNA on prognosis. However, adjustments for potential risk factors varied.

#### CtDNA mutations and DFS

Ryan et al. invested serum KRAS2 mutations in patients ranging from early to advanced CRC, and found that postoperative serum mutant KRAS2 was an independent factor of disease recurrence [7]. In fact, serum KRAS2 was stronger than the influence of Dukes’ stage or of treatment with adjuvant chemotherapy in the predict of CRC recurrence by a Cox regression multivariate analysis [7].

Tie et al. analyzed hotspot mutations in TP53, APC, KRAS, NRAS, BRAF, PIK3CA, CTNNB1, SMAD4, and FBXW7 in tumor tissue in CRC patients with stage II [32]. The identified mutations were then detected in plasma. An exploratory analysis of the correlation between ctDNA and clinicopathologic features showed that ctDNA was an independent factor, associated with a shorter recurrence-free survival (RFS) [32].

Lindforss et al. assessed the prognostic values of circulating KRAS in CRC patients with stagesI-III and found that no significant correlation between relapse of disease and KRAS mutation status in circulating DNA postoperatively on day three [25].

Wang et al. aimed to determined the presence of APC, KRAS, and P53 mutations in serum from CRC patients with Dukes’ stage A-D [28]. The correlation between the detection of ctDNA and the development of postoperative recurrence was significant (p<0.001) [28].

Tie et al. focused on TP53, APC, KRAS mutations in CRC patients with stage II [13]. They found that ctDNA detected postoperatively had a significantly reduced RFS (P<0.01). A multivariate analysis showed that ctDNA status was an independent variable to estimate the probability of RFS after adjustment for T stage, lymph node yield, and lymphovascular invasion [13].

#### CtDNA methylation and DFS

After controlling for the classic risk factors such as tumor size, lymph node status, and age at diagnosis, Herbst et al. showed that serum methylation of HLTF was associated with a high risk of disease recurrence, and serum methylation of HLTF proved to be an independent prognostic factor for patients with stages I-III in a multivariate analysis [27].

Lee et al. focused SEPT9 methylation (mSEPT9) in ctDNA among patients with stages I-II, and found that mSEP9 in ctDNA was significantly associated with lower DFS by univariate analysis. However, mSEP9 in ctDNA was not an independent prognostic factor in a multivariate analysis [24].

Tham et al. investigated the prognostic values of serum methylation of TAC1, SEPT9 and NELL1 among patients with stages I-II [16]. Their study showed that high serum methylation of TAC1 and SEPT9 but not NELL1 were independent predictors of unfavorable DFS after adjustment for vascular embolism, perineural invasion and serum CEA [16].

The detecting panel of Liu et al contained seven serum methylation markers: SST, MAL, TAC1, SEPT9, EYA4 and CRABP1 as well as NELL1 [20]. Only methylation of SST was found to be an independent predictor of unfavorable DFS in a multivariable Cox analysis, with adjustment for the effects of lymphovascular invasion, perineura invasion and serum CEA in combined group of stages II and III. When studying in individual subsets of stage II or III along, the researchers found that the statistical significance of serum methylation of SST was no longer retained [20].

Lin et al. explored the prognostic values of plasma AGBL4, FL11 and TWIST1 methylation in patients with stages I-IV. In univariate analysis, these three hypermathylated markers or serum GL11 hypermethylation were associated with poorer DFS (P<0.05). However, after stepwise elimination of tumor stage, lymphovascular invasion, preoperative CEA, mucinous histology and differentiation, none of these three markers were associated with patient outcome [19].

#### CtDNA mutation/methylation and DFS

Bazan et al. prospectively evaluated KRAS and TP53 mutations as well as p16^INK4A^ methylation status in primary CRC patients [23]. The univariate analysis showed that only KRAS mutations were associated with quicker relapse (P<0.01) [23]. No multivariate analysis was performed in their study.

### Relationship between ctDNA and OS

Thirteen studies assessing the relationship between ctDNA status and OS were eligible for the systematic review [6,8,9,12,14,17,18,20,21,23,26,29,34]. Eleven of them used the Kaplan-Meier method to estimate survival distribution curves [8,9,12,14,17,18,20,21,23,26,29]. Nine of 13 studies performed multivariate Cox regression analyses to explore the association between ctDNA status and OS [6,8,9,12,14,17,18,21,26].

#### CtDNA mutation and OS

Messaoudi et al. detected cfDNA *KRAS* and *BRAF* mutations in mCRC patients [34]. They found no statistic differences in OS between the *KRAS*-WT and *KRAS*-mutant mCRC patients. However, there was a statistically high significant difference between the median OS of *BRAF*-mutant patients and *BRAF*-WT patients. Compared to patients WT for *BRAF V600E*, *BRAF*-mutant mCRC patients had a shorter OS. A multivariate COX proportional hazards model showed that after controlling for CEA, tumor localization and age, *BRAF*-mutant status is a independent prognostic value (p = 0.002, HR = 7.33, 95% CI [1.04–2.89]) [34].

Spindler et al. came up with a quite different conclusion. They investigated the clinical implications of *KRAS* and *BRAF* mutations in ctDNA in patients with mCRC [9]. They found that *KRAS* mutations in ctDNA were independent prognostic factors and were associated with decreased OS by multivariate analysis, controlling for effects of age and performance status (PS). However, the effect of *BRAF* mutations in ctDNA on OS did not reach significance [9].

Sefrioui et al. analyzed *KRAS* mutations in ctDNA in patients with mCRC, and found that *KRAS* mutations in ctDNA were predictors of worse OS [29]. However, no multivariate analysis was performed.

Trevisiol et al. investigated the prognostic significance of circulating *KRAS2* mutations in patients with Dukes’ A, B, C, D [6]. Serum *KRAS2* mutations were significantly associated with a worse OS in multivariate analysis when adjusting for effects of CEA and stage [6].

Bai et al assessed the prognostic value of *KRAS* mutations in ctDNA in mCRC [12]. They classified ctDNA in a trichotomized manner (wild-type group, low-mutation group, and high-mutation group) and found that *KRAS* mutations were significantly associated with poorer prognosis by a multivariate analysis after adjusting for performance status and times of surgery as well as metastatic sites [12].

A panel of 74 genes were detected in circulating DNA in patients with stages I-III by Lin et al. [14]. They conducted a univariate analysis and found that the 5-year OS among patients who harbored a mutation in circulating DNA was significantly poorer than those without a ctDNA mutation. However, a multivariate analysis showed that the mutation status of ctDNA was no longer associated with patients’ survival when controlling for TNM stage, status of lymphovascular invasion and preoperative CEA level [14].

#### CtDNA methylation and OS

Leung et al. tested the role of serum methylation of *APC*, *hMHL1* and *HLTF* in CRC with stages I-IV [17], and found that none of these three markers individually were associated with OS. When combining these three markers together, there is a trend to a poor chance of OS in patients harboring any one of these three markers (p = 0.08) [17].

Liu et al. assessed the prognostic potentials of methylation *SST*, *NMV*, *MAL*, *TAC1*, *SEPT9* and *EYA4* in the serum of CRC patients with stages I-IV [20]. Univariate analyses showed that serum methylation levels of *MAL* and *SST* were significantly predictive of cancer-specific death. Multivariate Cox regression model revealed the independent prognostic effect of serum methylation of *SST* on OS, adjusting for stage, perineural invasion, lymphovascular invasion [20]. The effects of serum methylation of *SST* were also investigated based on tumor stages. In the combined group of stages II and III, serum *mSST* remained as a significant independent predictor of worse OS (HR = 2.797, 95% CI, 1.34–5.84; *P* = 0.006) [20]. When analyzed in patients with each stage individually, independent predictive effect of serum *mSST* on OS remained significance only in stage III subgroup (HR = 2.52, 95% CI, 1.02–6.25; *P* = 0.045) [20].

Philipp et al. focused on serum methylation of *HLTF* and *HPP1* in CRC patients with stages I-IV [26]. Their results showed that the presence of *HLTF* or *HPP1* was significantly associated with poorer prognosis [26]. The researchers then examined the prognostic values of serum *HLTF* and *HPP1* methylation in subgroup analyses according to tumor stage. Serum *HLTF* but not *HPP1* methylation was associated with shorter survival in patients with stage I. Neither serum *HLTF* or *HPP1* methylation was associated with OS in patients with stages II and III or in patients with the combined stages I-III. In patients with stage IV, serum *HLTF* and *HPP1* methylation conferred a significantly worse OS. A multivariate analysis showed that the methylation of *HLTF* and the methylation of *HPP1* were independent prognostic factors in patients with stage IV, whereas controlling for effects of CEA.

Wallner et al. studied the prognostic potential of ctDNA methylation in CRC patients with stage I-III [18]. Three genes *HPP1*/*TPEF*, *HLTF* and *hMLH1* were studied to assess their prognostic effects on OS. The multivariate analysis showed that *HPP1*/*TPEP* and *HLTF* along or in combination were independent prognostic factor of worse OS after adjusting for lymph node metastases, distant metastases, age, tumor size [18].

Matthaios et al. investigated the prognostic value of the methylation status of *APC* and *RASSF1A* in ctDNA in patients with early operative CRC and metastatic CRC [21]. Multivariate Cox proportional hazards regression analysis was performed to investigate the independent effects of methylated APC and *RASSF1A* promoter status on OS, adjusting for patients’ gender, age, clinical stage, tumor differentiation, lymph node status, CEA and CA199 levels. The results showed that methylated *APC* promoter status have a significant negative impact on the survival of patients with (adjusted HR = 3.47, 95% CI 1.35–8.92, P = 0.017) or without metastases (adjusted HR = 7.88, 95% CI = 2.73–22.73, P<0.001). Methylated *RASSF1A* promoter status was also associated with a worse survival in patients with (adjusted HR = 5.76, 95% CI 2.44–14.82, P = 0.001) or without metastases (adjusted HR = 3.06, 95% CI 1.25–7.50, P = 0.038)(21).

#### CtDNA mutation/methylation and OS

Lecome et al. focused on *KRAS2* mutations and *p16* hypermethylation in CRC patients with stages I-III [8]. For OS, patients with dateable ctDNA had a significantly worse survival than those without detectable ctDNA by the univariate analysis. When adjusting for TNM’s stage, the multivariate analysis revealed that this significance disappeared [8].

Bazan et al. investigated whether the detection of *TP53*, *KRAS* mutations and *p16*^*INK4A*^ methylation in ctDNA was associated with OS [23]. Their results showed that no significant association had been found between these alterations in ctDNA and OS [23].

## Discussion

To the best of our knowledge, this is the first systematic review to explore the relationship between ctDNA status and prognosis in patients with CRC.

Most papers included in our review found that ctDNA was associated with a worse DFS/OS in patients with CRC. Some of them declared that ctDNA had an independent significant effect on patients’ prognosis. Some showed that not all of genetic/epigenetic alterations detected in ctDNA in their detection panels were correlated with patients’ prognosis. A few studies showed that the prognostic effects of ctDNA could only be found in certain patients, such as patients with advanced stages. The divergent conclusions of the included papers may result from detection panels, patients, detection methods, matrixes (plasma or serum), treatment and sampling time.

In our systematic review, one of the major confounding factors was the absence of a standardized definition of ctDNA. Additionally, there was no consensus regarding the detecting panel, marker threshold, detection method, time of sample collection, or sample type. The included studies revealed that both genetic and epigenetic alterations could be detected in ctDNA in patients with CRC. It was reported that certain genetic alterations such as *KRAS* mutations in ctDNA were highly specific for colorectal neoplasia. Compared with genetic alterations, epigenetic alterations appeared to be less specific as tumor markers. Epigenetic alterations could also be detected in non-tumor cells, and their frequency may increase in older patients [35]. Additionally, the frequency of methylation varies in different suppressor tumor genes and changes with tumor stage. In most studies, aberrant hypermethylation of ctDNA was found to be associated with a worse prognosis. However, contradicting results were also reported by other studies because of the use of different detecting panels. Because of a lack of a recommended standard marker panel, most studies chose markers that had been reported to occur more frequently in patients with CRC than in healthy patients. In our included studies, the number and type of alterations varied. Hence, in future studies, a standard marker panel with both satisfactory sensitivity and specificity will be needed.

The low level of ctDNA is another challenge for successful detection. In early studies, ctDNA alterations were in low agreement with those in tumor tissue because of the low levels of ctDNA. For example, a study by Lecomte et al. found that serum alterations could be detected in less than half of the patients harboring tissue alterations [8]. The discordance in the alteration detection rate between tumor tissue and plasma or serum could be resolved by high-sensitivity detection methods. In our included studies, the detection sensitivity varied according to the detection method. Regarding *KRAS* mutation detection, for example, the sensitivity and specificity of next-generation sequencing-based SafeSeq technology were 87.2 and 99.2%, respectively, whereas for the optimized clamp-PCR Intplex test, the sensitivity and specificity were 92 and 98%, respectively [5,36]. Droplet-based PCR had a sensitivity of 80%. In chip-based digital PCR, only 69% of *KRAS*-positive patients were detected [29], whereas ARMS-based PCR methodology could detect a mutant gene with a frequency as low as 0.1% [37]. Inevitably, a low-sensitivity method may miss low amounts of tumor-associated alterations in circulating. Data should be interpreted with caution because low sensitivity methods can only detect ctDNA in patients if present at a high level.

Sampling time also has an important effect on the successful detection of ctDNA. CtDNA dynamically changes during therapy [38]. It has been reported that previously detected mutations are undetectable one week after surgery. In chemotherapy-responsive patients, ctDNA levels can decrease or increase to detectable levels [39,40]. Tumor-associated alterations in circulating DNA may emerge during the follow-up period [7]. In vivo, ctDNA has a short half-life. Its appearance indicates that an occult tumor exists after therapy. Another aspect that needs to be addressed is clonal selection. Clonal selection is normally induced by treatment and causes the ratio between wild-type and mutated cells to change over time [41]. Taking these variables into consideration, the timing of the blood sample collection is critical for successful detection. Hence, it is necessary to continuously collect blood samples from patients both before and at least 1 month after therapy.

In the eligible studies, either serum or plasma was used for ctDNA detection. Strictly speaking, the DNA content of serum and plasma can be drastically different. Serum samples contain higher total free DNA yields, perhaps because of the release of DNA caused by cell lysis during coagulation. This large amount of non-tumor DNA released by normal cells in serum means that the fraction of tumor DNA in serum is lower than that in plasma. This difference may affect ctDNA detection.

Our comprehensive review revealed that a ctDNA-positive status is associated with a worse prognosis. However, some limitations should be considered when interpreting our findings. The major limitations included clinical and methodological heterogeneity. Although some studies excluded patients with familial adenomatous polyposis or hereditary non-polyposis colorectal cancer [6,15], the detection panels varied across the studies. Additionally, the different follow-up times, detection methods, and intrinsic differences in treatment regimens and blood sample collection times also contributed to the heterogeneity. Some included studies only analyzed ctDNA status in patients with tissue alterations [8,15]. Some studies included in our review investigated the prognosis effects of ctDNA by a multivariate analysis. However, the confounders adjusted for also differed between studies. To include the maximal number of relevant studies, we treated RFS and DFS synonymously, knowing that a small percentage of patients with secondary primary cancers could be included.

The current gold standards in determining prognosis in CRC are Dukes’ staging and TNM staging [42]. However, for patients in intermediate groups (stage II-III tumors), these staging systems are less informative. Until now, only the serum biomarker carcinoembryonic antigen has been confirmed to provide prognostic information in Dukes’ B, or an equivalent, stage of CRC. One included study showed that after adjustment for other significant factors, the correlation between ctDNA and prognosis was still strong, stronger than Dukes’ stage of disease [7].

Reports illustrated that tissue genotyping could contribute to prognostic and predictive biomarker evaluation in patients with CRC [43–45]. Because of inherent tumor heterogeneity and invasive sampling, tumor tissue genotyping has limitations. Other studies suggested that the primary tumor has a different genomic landscape than metastases. Additionally, the invasive nature of the procedure means continuous sampling cannot be performed. Mutated DNA in circulating is of tumor origin. It was reported that plasma mutations have better prognostic value than tumor tissue mutations [9]. Lecomte et al. found that among patients with tissue alterations, ctDNA alteration-positive patients had a worse prognosis [8]. Furthermore, a study by Philipp et al. uncovered that the presence of ctDNA was correlated with carcinoembryonic antigen expression and that ctDNA status was a stronger prognostic biomarker than tumor stage in CRC. These studies shed some light on the prognostic value of ctDNA in CRC.

In summary, this is the first comprehensive analysis to assess the prognostic value of ctDNA in patients with CRC using the currently available literature. The findings of this systematic review strongly suggest that patients with ctDNA-positive CRC have an unfavorable prognosis. In view of some of the limitations of this study, we conclude that a large cooperative study is needed to address the prognostic and predictive value of ctDNA in patients with tumors.

## Supporting information

S1 Fig Bias graph: Each risk of bias item presented as a percentage across all included studies. (TIFF)

S1 Table Reasons for exclusion after examining the full text. (DOCX)

S2 Table Additional details related to the patient inclusion criteria, pre-analytical and analytical characteristics of the implemented assay. (DOCX)

S3 Table PRISMA checklist. (DOC)
