# Correction: Prognostic value of circulating tumor DNA in patients with colon cancer: Systematic review

**DOI:** 10.1371/journal.pone.0338537

**Published:** 2025-12-15

**Authors:** Gaowei Fan, Kuo Zhang, Xin Yang, Jiansheng Ding, Zujian Wang, Jinming Li

Following publication of this article [1], errors were identified in Fig 1, Table 1, and the Methods section. Specifically,

- The ‘Records excluded’ box of Fig 1 incorrectly shows ‘n = 1839’; the correct value is ‘n = 1883’.
- The publication year of the ‘Lin (2016)’ citation in the bottom row of Table 1 is incorrect; the correct citation is ‘Lin (2015)’.
- There is an error in the third sentence of the Data extraction subsection of the Methods section. The correct sentence is: Data extraction was performed by four independent investigators with a predefined information sheet (Gaowei Fan, Kuo Zhang, Xin Yang, Jiansheng Ding).

Corrected versions of Fig 1 and Table 1 are provided with this notice.

**Fig 1:**
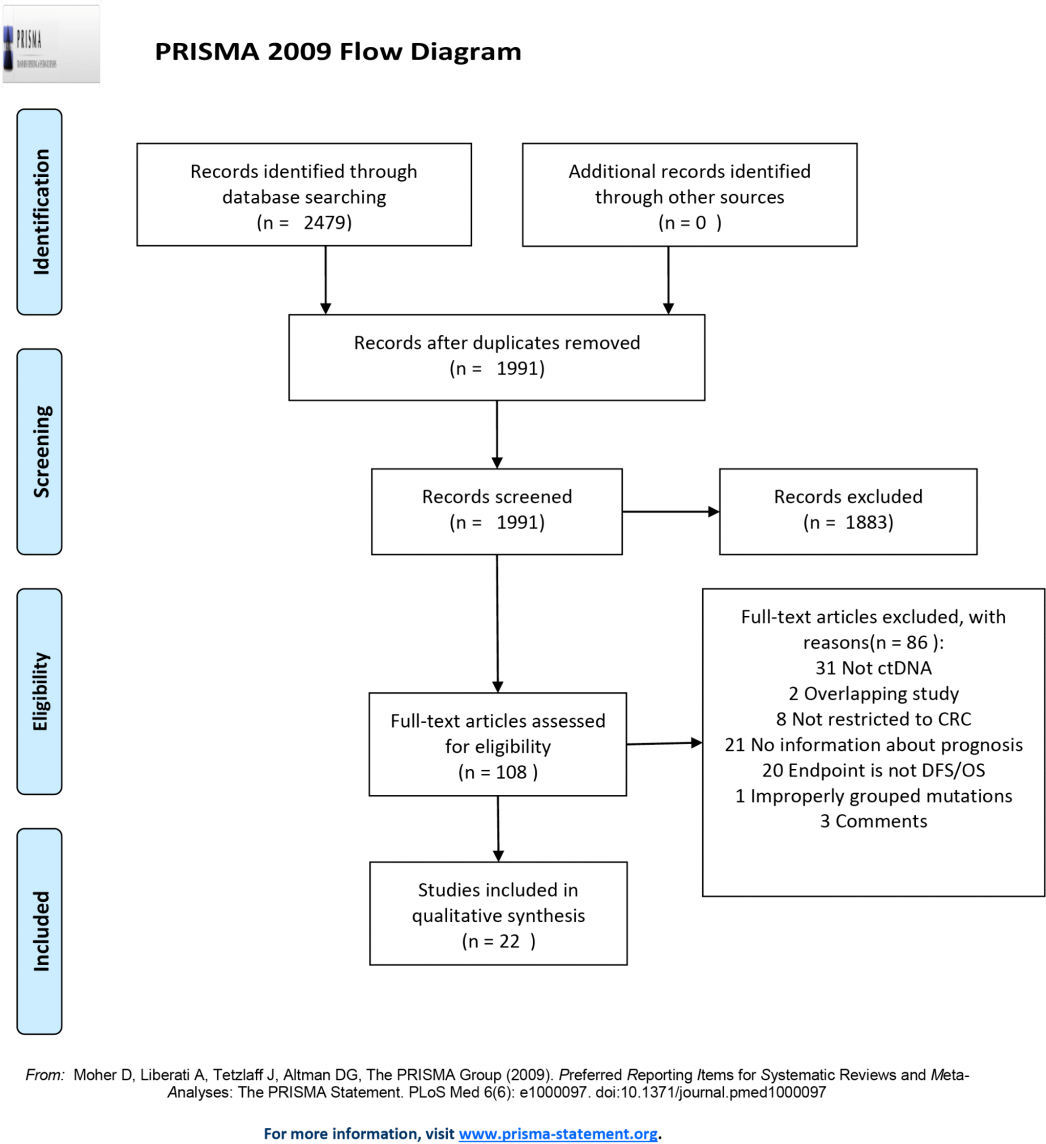
PRISMA 2009 flow diagram. PRISMA flow diagram for study selection. *From*: Moher D, Liberati A, Tetzlaff J, Altman DG, The PRISMA Group (2009). *P*referred *R*eporting *I*tems for *S*ystematic Reviews and *M*eta-*A*nalyses: The PRISMA Statement. PLoS Med 6(6): e1000097. doi:101371/journal.pmed.1000097. For more information, visit www.prisma-statement.org.

**Table 1:** General characteristics of the study populations.

First author name (year)	Country	Publication type	Study design	Patients included, n	ctDNA-positive patients, n	Male (%)	Tumor stage	Median age	ctDNA panel	Detection methods	Study treatment	Sampling time	Median follow-up	Outcome
Lecomte (2002)	France	FP	PRO	37	26	NR	TNM I-IV	NR	*KRAS2*, *p16*	MASA, MSP	Sur, chem, rad	Pre-tr	22 months	OS
Spindler (2013)	Denmark	FP	PRO	97	30^KRAS^,8^BRAF^	65.6%	metastasis	66	*KRAS2*, *Braf*	ARMS-qPCR	Irinotecan, mono	Pre-tr,	NR	OS
Trevisiol (2006)	Italy	FP	PRO	15	7	NR	Dukes’ A, B, C, D	NR	*KRAS2*	ME-PCR	Sur	Pre-tr	41 months	OS
Ryan (2003)	The Netherlands	FP	PRO	85	16	NR	Dukes’ A, B, C	NR	*KRAS2*	SN-PCR, DS	Sur	Post-tr	3 years	DFS
Bai (2013)	China	FP	Retro	106	17^low mutation^, 16^high mutation^	56.6%	metastasis	55.5	*KRAS*	PNA-PCR, nested PCR	Sur, chem, cetuximab	NR	21.3 months	OS
Messaoudi (2016)	France	FP	PRO	97	38^KRAS^, 5^BRAF^	59.8%	metastasis	66.6	*KRAS, BRAF*	Intplex	Chemo, rad	Pre-tr	36 months	OS
Sefrioui (2015)	France	FP	PRO	16	11	37.5%	metastasis	NR	*KRAS2*	chip-based digital PCR	Chem	NR	NR	OS
Tie (2014)	America	Meeting	PRO	78	6	NR	TNM II	66	*TP53*, *APC*, *KRAS*, *NRAS*, *BRAF*, *PIK3CA*, *CTNNB1*, *SMAD4*, *FBXW7*	Safe-SeqS	Sur, chem	NR	2 years	RFS
Tie (2016)	Australia	FP	PRO	230	20	57%	TNM II	65	*TP53, APC, KRAS*	Safe-SeqS	Sur with chem or not	Post-tr	27 months	RFS
Bazan (2006)	Italy	FP	PRO	50	8^KRAS^, 8^TP 53^	NR	Primary	NR	*TP53 KRAS p16INK4A*	SSCP-PCR, MSP	Sur	NR	26 months	DFS, OS
Lin (2014)	Taiwan	FP	PRO	133	41	41.3%	TNM I-IV	NR	74 genes	MassArray	Sur	Pre-tr	62 months	OS
Lindforss (2005)	Sweden	FP	PRO	25	9	36%	TNM I-III	72	KRAS	TGGE	Sur	Pre-tr &Pro-tr	38 months	DFS
Wang (2004)	Taiwan	FP	PRO	104	36	48.7%	Dukes’ A, B, C, D	DD62.1; DU65.9	36	PCR-SSCP	Sur	Pre-tr	20 months	DFS
Herbs t(2009)	Germany	FP	Retro	106	13	NR	UICC I-III	66	*HLTF*, *HPP1*, *TPEF*	MethyLight	Sur	Pre-tr	5 years	RFS
Lee (2013)	Korea	FP	PRO	101	37	32.7%	TNM I-IV	NR	*SEPT9*	Real-time PCR	Sur, chem, rad	Pre-tr	518 days	DFS
Leung (2005)	Hong Kong	FP	PRO	49	28	36.7%	TNM I-IV	57	*APC*, *hMLH1*, *HLTF*	MethyLight	Any treat	Pre-tr	13.6 months	OS
Philipp (2012)	Germany	FP	PRO	311	48^HLTF^; 64^HPP1^	55%	TNM IV	NR	*HLTF*, *HPP1*	MSP	NR	Pre-tr,	8 years	OS
Tham (2014)	Singapore	FP	Retro	150	NR	56.7%	TNM I-II	NR	*TAC1*, *SEPT9*, *NELL1*	MSP	Sur	Pre-tr,	59 months	DFS
Wallner (2006)	Germany	FP	Retro	77	20	NR	TNM I-III	NR	*HPP1*, *HLTF*	MethyLight	NR	Pre-tr	5 years	DFS
Matthaios (2016)	Greece	FP	PRO	155	22^RASSF1A^, 29^APC^	57.4%	Dukes’ A,B,C,D	70	*RASSF1A, APC*	MSP	Sur	Pre-tr	NR	OS
Liu (2016)	Singapore	FP	PRO	165	82	55.2%	TNM I-IV	67	*SST*	MSP	Sur	Pre-tr	56 months	DFS, OS
Lin (2015)	Taipei	FP	PRO	353	129	60.06%	TNM I-IV	67	*AGBL4, FLI1, TWIST1*	Sequenom MassCLEAVE and MALDI-TOF	Sur	Pre-tr	56 months	DFS

PRO, prospective study; retro, retrospective study; FP, full publication article; pt, patient; NR, no report; chem, chemotherapy; FU, follow-up; pre-tr, pretreatment; sur, surgery; rad, radiotherapy; mono, monotherapy; meeting, ASCO meeting abstract; post-tr, after treatment; DS, direct sequencing; PCR, polymerase chain reaction; MASA, mutant allele-specific amplification; MSP, methylation-specific PCR; ARMS-qPCR, allele refractory mutation systems-based quantitative PCR; ME-PCR, mutant-enriched PCR; SN-PCR, semi-nested PCR; SSCP-PCR, single-strand conformation polymorphism-PCR; RFS, recurrence-free survival; OS, overall survival; DFS, disease-free survival; SSCP-PCR, single-strand conformation polymorphism-PCR; MALDI-TOF, matrix-assisted laser desorption ionization–time of flight mass spectrometry; TGGE, temperature gradient gel electrophoresis; DD, circulating DNA-detectable; DU, circulating DNA-undetectable.
