# Supplementary figures and images for: Prognostic value of circulating tumor DNA in patients with colon cancer: Systematic review
Source: PLoS One. 2017 Feb 10;12(2):e0171991. doi: 10.1371/journal.pone.0171991 (PMC5302475; doi:10.1371/journal.pone.0171991)

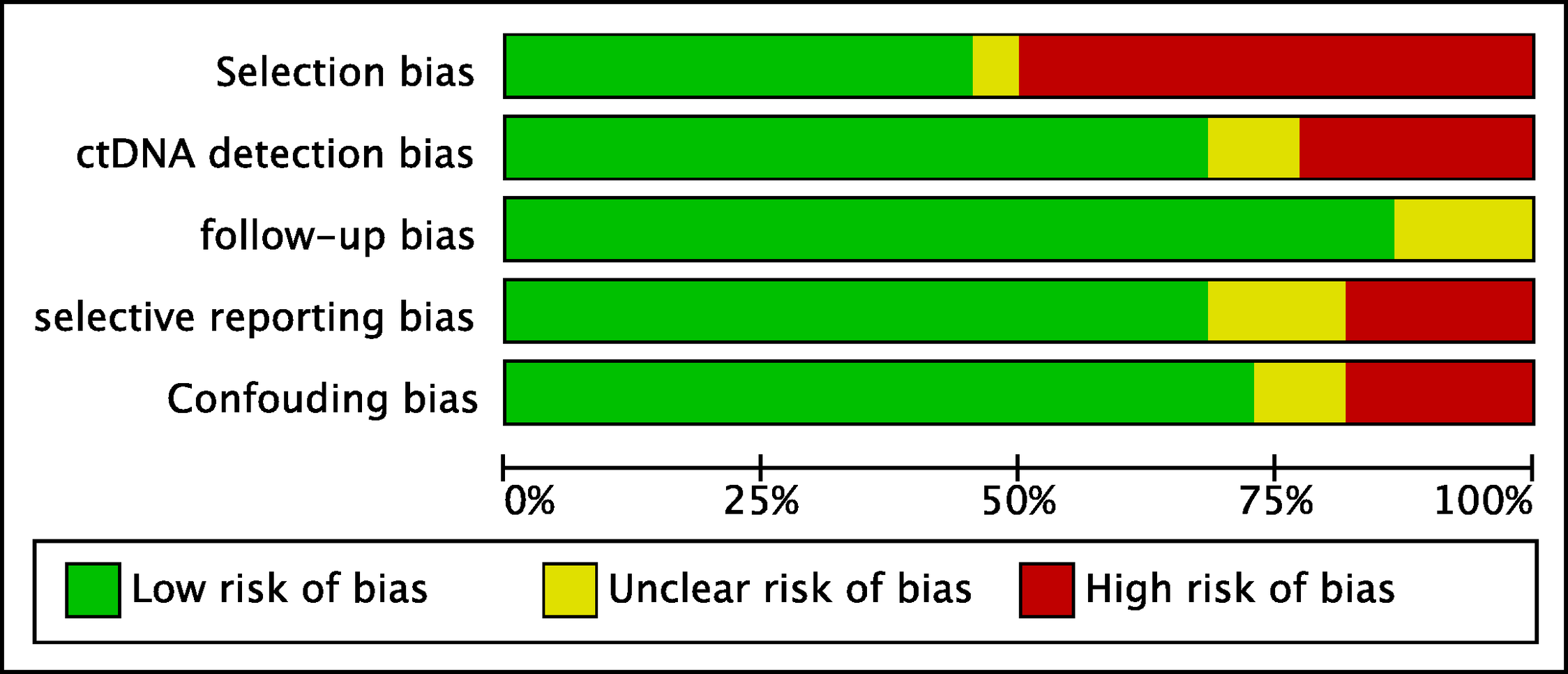

Supplement: S1 Fig — (TIFF) [file pone.0171991.s001.tiff]
