# Supplementary material for: Prognostic value of circulating tumor DNA in patients with colon cancer: Systematic review
Source: PLoS One. 2017 Feb 10;12(2):e0171991. doi: 10.1371/journal.pone.0171991 (PMC5302475; doi:10.1371/journal.pone.0171991)
Supplement: S1 Table — (DOCX) [file pone.0171991.s002.docx]

Reasons for exclusion after examining the full text:

| NO. | Title | Reasons for exclusion |
| --- | --- | --- |
| 1 | Tumour pharmacodynamics and circulating cell free DNA in patients with refractory colorectal carcinoma treated with regorafenib | The outcome is PFS |
| 2 | Tumor MGMT promoter hypermethylation changes over time limit temozolomide efficacy in a phase II trial for metastatic colorectal cancer | Using FFPE tissue samples for gene alterations detecting |
| 3 | Digital PCR quantification of MGMT methylation refines prediction of clinical benefit from alkylating agents in glioblastoma and metastatic colorectal cancer | The outcome is PFS |
| 4 | Adverse prognostic impact of the CpG island methylator phenotype in metastatic colorectal cancer | Using tumor tissue samples for gene alterations detecting |
| 5 | Evaluation of CpG Island Methylator Phenotype as a Biomarker in Colorectal Cancer Treated With Adjuvant Oxaliplatin | DNA and total RNA were extracted from 1.0-mm tissue microarray cores |
| 6 | Methylation of MGMT Is Associated with Poor Prognosis in Patients with Stage III Duodenal Adenocarcinoma." | Genomic DNA was extracted from FFPE tissues. |
| 7 | Diagnostic and prognostic value of SHOX2 and SEPT9 DNA methylation and cytology in benign, paramalignant, and malignant ascites. | DNA methylation of SHOX2 and SEPT9 were detected by using the cellular fraction of the ascites |
| 8 | Can Serum be Used for Analyzing the KRAS Mutation Status in Patients with Advanced Colorectal Cancer? | DNA was extracted from five paraffin sections of 10-μm thickness containing a representative portion of the tumor tissue |
| 9 | Mismatch repair deficiency predicts benefit of anti-PD-1 therapy. | Comments |
| 10 | PD-1 blockade in tumors with mismatch-repair deficiency | Mismatch-repair status was assessed in tumors |
| 11 | LINE-1 Methylation Status Correlates Significantly to Post-Therapeutic Recurrence in Stage III Colon Cancer Patients Receiving FOLFOX-4 Adjuvant Chemotherapy. | DNA was detracted from formalin-fixed, paraffin-embedded (FFRE) specimens |
| 12 | High RAD54B expression: an independent predictor of postoperative distant recurrence in colorectal cancer patients | Total RNA was extracted from tissue samples using TRIzol Reagent |
| 13 | Prognostic significance of PLA2G4C gene polymorphism in patients with stage II colorectal cancer. | Genomic DNA was isolated from the lymphocytes |
| 14 | Selecting and Combining Biomarkers by Gene Expression Profiles for Colon Cancer Classification. | Gene mutations were detected by using tumor tissue |
| 15 | Genome Wide Association Study for Predictors of Progression Free Survival in Patients on Capecitabine, Oxaliplatin, Bevacizumab and Cetuximab in First-Line Therapy of Metastatic Colorectal Cancer | Whole blood was collected at baseline and germline DNA was isolated from peripheral leukocytes |
| 16 | Methylation status at HYAL2 predicts overall and progression-free survival of colon cancer patients under 5-FU chemotherapy | DNA were isolated from needle biopsies with 0.6 mm in diameter from FFPE tumor tissue |
| 17 | DNA repair gene XPG C46T polymorphism predicts response to platinum-based neoadjuvant chemoradiotherapy and prognosis in clinical stage II/III rectal cancer patients | Genomic DNA was obtained from peripheral blood |
| 18 | Prognostic and predictive value of extended RAS mutation and mismatch repair status in stage III colorectal cancer | DNA was extracted from formalin-fixed, paraffin-embedded tumor samples. |
| 19 | Molecular markers identify subtypes of stage III colon cancer associated with patient outcomes | Mutation status was determined using genomic DNA extracted from macrodissected, formalin-fixed, paraffin- embedded tumor tissue |
| 20 | Variations in genes involved in immune response checkpoints and association with outcomes in patients with resected colorectal liver metastases | Genomic DNA from formalin-fixed paraffin-embedded CLM was extracted |
| 21 | Predictive impact of genetic polymorphisms in DNA repair genes on susceptibility and therapeutic outcomes to colorectal cancer patients | Genomic DNA was isolated from a leukocyte cell pellet of each blood sample |
| 22 | DNA Hypermethylation of SHISA3 in Colorectal Cancer: An Independent Predictor of Poor Prognosis | Mutations were detected by using cell lines |
| 23 | Phase I study of azacitidine and oxaliplatin in patients with advanced cancers that have relapsed or are refractory to any platinum therapy | Methylation studies were done using biopsy samples. |
| 24 | KRAS codon 12 and 13 mutations in relation to disease-free survival in BRAF-wild-type stage III colon cancers from an adjuvant chemotherapy trial (N0147 alliance) | both KRAS and BRAF mutation status were determined using DNA extracted from macrodissected formalin-fixed, paraffin-embedded tumor tissue |
| 25 | Combined detection of plasma GATA5 and SFRP2 methylation is a valid noninvasive biomarker for colorectal cancer and adenomas | No information about DFS or OS. |
| 26 | Circulating free DNA, mutation detection and predictive biomarker potential | No information about DFS or OS. |
| 27 | Changes in Colorectal Carcinoma Genomes under Anti-EGFR Therapy Identified by Whole-Genome Plasma DNA Sequencing | No information about DFS or OS |
| 28 | Oncogenic transformation induced by cell-free nucleic acids circulating in plasma (genometastasis) remains after the surgical resection of the primary tumor: a pilot study | No information about DFS or OS |
| 29 | Detection of tumor-associated mutations in circulating DNA: Clinical applications and experiences | No information about prognosis |
| 30 | comment on ‘KRAS-mutated plasma DNA as predictor of outcome from irinotecan monotherapy in metastatic colorectal cancer’ | Comments |
| 31 | ctDNA is a specific and sensitive biomarker in multiple human cancers. | No information about prognosis |
| 32 | Actionable Mutations in Plasma Cell-Free DNA in Patients with Advanced Cancers Referred for Experimental Targeted Therapies | Selected tumors were not restricted to CRC. |
| 33 | Mutation profiling of tumor DNA from plasma and tumor tissue of colorectal cancer patients with a novel, high-sensitivity multiplexed mutation detection platform | No information about prognosis |
| 34 | Detection of Circulating Tumor DNA in Early- and Late-Stage Human Malignancies | Selected tumors were not restricted to CRC |
| 35 | ctDNA is a specific and sensitive biomarker in multiple human cancers | Selected tumors were not restricted to CRC |
| 36 | Detection of mutant circulating tumor DNA (CTDNA) in plasma of patients with colorectal cancer (CRC) | No information about prognosis |
| 37 | Quantitative analysis of plasma DNA in colorectal cancer patients: a novel prognostic tool | Not ctDNA |
| 38 | Targeted deep sequencing from circulating plasma DNA as a multipurpose biomarker in pts (pts) referred for phase I trials | The selected cancers were not restricted to CRC. |
| 39 | APC, K-ras, and p53 genc mutations in colorectal cancer patients: correlation to clinicopathologic features and postoperativie survieillance. | No information regarding DFS or OS |
| 40 | Actionable mutations in plasma cell-free DNA in patients with advanced cancers referred for experimental targeted therapies | The selected cancers were not restricted to CRC. |
| 41 | Utility of cell-free tumour DNA for post-surgical follow-up of colorectal cancer patients | No information regarding DFS or OS |
| 42 | Blood-based treatment monitoring of metastatic colorectal cancer patients by KRAS mutation analysis | No information regarding DFS or OS |
| 43 | KRAS mutation analysis using blood derived cell free tumor DNA for treatment monitoring of metastatic colorectal cancer patients | No information regarding DFS or OS |
| 44 | Changes in Colorectal Carcinoma Genomes under Anti-EGFR Therapy Identified by Whole-Genome Plasma DNA Sequencing | No information regarding DFS or OS |
| 45 | Characterizing the patterns of clonal selection in circulating tumor DNA from patients with colorectal cancer refractory to anti-EGFR treatment | The outcome is PFS |
| 46 | Prospective study of oncogenic mutations in circulating cell-free DNA (cfDNA) using a multiplex sequencing platform for patient (pt) allocation to phase I clinical trials | Did not investigated the relationship between ctDNA and prognosis. |
| 47 | Cell-free DNA, KRAS, and BRAF mutations in plasma from patients with metastatic colorectal cancer treated with third-line cetuximab and irinotecan | KRAS mutations were divided into high (>75% percentile) and low (<75% percentile) groups. |
| 48 | Multi-purpose utility of circulating plasma DNA testing in patients with advanced cancers | The selected tumors were not restricted to CRC |
| 49 | Circulating free DNA in a screening program for early colorectal cancer detection | No information regarding DFS and OS |
| 50 | Clinical value of chip-based digital-PCR platform for the detection of circulating DNA in metastatic colorectal cancer | Not ctDNA |
| 51 | Plasma cell-free DNA and fraction of circulating KRAS mutations as prognostic in patients with metastatic colorectal cancer | Comparing different treatment |
| 52 | Circulating free DNA as biomarker and source for mutation detection in metastatic colorectal cancer | No information about the effects of ctDNA on prognosis |
| 53 | Response to comment on 'KRAS-mutated plasma DNA as predictor of outcome from irinotecan monotherapy in metastatic colorectal cancer' | Comments |
| 54 | Circulating free DNA as biomarker and source for mutation detection in metastatic colorectal cancer | No information about the effects of ctDNA on prognosis |
| 55 | Massively parallel sequencing (MPS) of circulating DNA in patients with metastatic colorectal cancer (mCRC): Prognostic significance and early changes during chemotherapy | No information about the effects of ctDNA on prognosis |
| 56 | Serum detection of KRAS c.35G>T (G12V) and c.35G>A (G12D) mutations in colorectal cancer patients by COLD-PCR HRM | No information regarding prognosis |
| 57 | Circulating free tumor DNA as a promising marker for the prediction of survival and monitoring of remission in colorectal cancer after radical surgery. | No information regarding prognosis |
| 58 | Circulating Free Tumor DNA as a Promising Marker for the Prediction of Survival and Monitoring of Remission in Colorectal Cancer After Radical Surgery | No information regarding DFS and OS |
| 59 | Identification of a DNA methylation signature to predict disease-free survival in locally advanced rectal cancer | No information regarding prognosis |
| 60 | A phase II study of combination epigenetic therapy in metastatic colorectal cancer (mCRC): A phase II consortium (P2C)/Stand Up 2 Cancer (SU2C) study | No information regarding prognosis |
| 61 | Aberrant Methylation of APC, MGMT, RASSF2A, and Wif-1 Genes in Plasma as a Biomarker for Early Detection of Colorectal Cancer | No information regarding prognosis |
| 62 | Prospective evaluation of methylated SEPT9 in plasma for detection of asymptomatic colorectal cancer | No information regarding prognosis |
| 63 | Identification of a DNA methylation signature to predict disease-free survival in locally advanced rectal cancer | Not ctDNA |
| 64 | Methylation and microsatellite status and recurrence following adjuvant FOLFOX in colorectal cancer | Not ctDNA |
| 65 | Methylation of TFPI2 no longer detected in the serum DNA of colorectal cancer patients after curative surgery | No information regarding prognosis |
| 66 | Methylated DAPK and APC promoter DNA detection in peripheral blood is significantly associated with apparent residual tumor and outcome | No information regarding prognosis |
| 67 | Tumor suppressor gene hypermethylation as a predictor of gastric stromal tumor behavior | Not CRC |
| 68 | CpG island methylator phenotype infers a poor disease-free survival in locally advanced rectal cancer | No information regarding DFS and OS |
| 69 | Hypermethylated APC DNA in plasma and prognosis of patients with esophageal adenocarcinoma | Not CRC |
| 70 | Promoter Methylation of Specific Genes is Associated with the Phenotype and Progression of Colorectal Adenocarcinomas | The detecting matrix is tumor tissue |
| 71 | Detection of colorectal adenoma and cancer by blood-based analysis of DNA methylation | No prognosis |
| 72 | TFAP2E Methylation Status and Prognosis of Patients with Radically Resected Colorectal Cancer | TFAP2E methylation status was detected by using tumor tissue. |
| 73 | Aberrant methylation of NPY, PENK, and WIF1 as a promising marker for blood-based diagnosis of colorectal cancer | No information regarding prognosis |
| 74 | Genetic and epigenetic classifications define clinical phenotypes and determine patient outcomes in colorectal cancer | Genomic DNA was isolated from frozen tissue |
| 75 | Association of CHFR Promoter Methylation with Disease Recurrence in Locally Advanced Colon Cancer | DNA methylation levels were measured using surgical specimens |
| 76 | Detection of RASSF1A promoter hypermethylation in serum from gastric and colorectal adenocarcinoma patients | No information regarding DFS and OS |
| 77 | MTHFR, MTR, and MTRR Polymorphisms in Relation to p16INK4A Hypermethylation in Mucosa of Patients with Colorectal Cancer | Genomic DNA was extracted from blood cells |
| 78 | Combined detection of plasma GATA5 and SFRP2 methylation is a valid noninvasive biomarker for colorectal cancer and adenomas | No information regarding prognosis |
| 79 | Detection of aberrant p16 methylation in the serum of colorectal cancer patients | No information regarding prognosis |
| 80 | Prognostic value of circulating mutant DNA in unresectable metastatic colorectal cancer | The outcome is PFS |
| 81 | Circulating mutant DNA to assess tumor dynamics | No information regarding DFS and OS |
| 82 | Plasma cell-free DNA and fraction of circulating KRAS mutations as prognostic in patients with metastatic colorectal cancer | Overlapping reports |
| 83 | KRAS mutated plasma DNA as predictor of outcome from irinotecan monotherapy in metastatic colorectal cancer | Overlapping reports |
| 84 | RUNX3 Promoter Methylation in Colorectal Cancer: Its Relationship with Microsatellite Instability and its Suitability as a Novel Serum Tumor Marker. | No information regarding DFS and OS |
| 85 | Epigenetic Inactivation of the BRCA1 Interactor SRBC and Resistance to Oxaliplatin in Colorectal Cancer | Not ctDNA |
| 86 | Quantitative Measurement of Cell-Free Plasma DNA and Applications for Detecting Tumor Genetic Variation and Promoter Methylation in a Clinical Setting | No prognosis |
