# Supplementary material for: Prognostic value of circulating tumor DNA in patients with colon cancer: Systematic review
Source: PLoS One. 2017 Feb 10;12(2):e0171991. doi: 10.1371/journal.pone.0171991 (PMC5302475; doi:10.1371/journal.pone.0171991)
Supplement: S2 Table — (DOCX) [file pone.0171991.s003.docx]

| Study name | patients inclusion | pre-analytical | | | | Analytical characteristic | | | | |
| --- | --- | --- | --- | --- | --- | --- | --- | --- | --- | --- |
|  |  | type of matrix: serum or plasma | Handling of blood samples between drawing and processing | Handling of samples between blood processing and nucleic acid extraction | ctDNA extracts | analytic assays | analytic specificity | analytic sensitivity | control | cut-off and quantification |
| lecomte 2002 | Patients underwent primary colorectal cancer resction were included. The tumor stage was TNMⅠ-Ⅳ. 4 patients with rectal cancer underwent preoperative radiotherapy. 16 patients received adjuvant chemotherapy. only patients for which  genetic alterations were previously found in tumors were selected. | plasma | Peripheral blood samples were  collected immediately before surgical resection into an EDTA  tube. | Blood samples were centrifuged for 10 min at 3,000g, to separate buffy coats and plasma. Plasma samples were stored at −80 ◦C prior to DNA extraction. | Plasma DNA was extracted using the QIAmp Blood Kit  (Qiagen, Courtaboeuf, France)  .Two milliliters of plasma were used, and a DNA  elution volume of 100 ul was obtained after extraction. In a second  step, DNA was concentrated in a final volume of 30 ul. | MASA was used to detect KRAS mutation. |  | Serial dilutions of SW480, a cell line that bears a G12D KRAS2 mutation, demonstrated that the MASA assays as described here were very sensitive as 1 mutant allele out of 1,000 normal alleles could be detected. The sensitivity of the method was tested  and 1 methylated genome in 500 unmethylated genomes could be  detected. | Negative  and positive controls were performed for each PCR reaction. The MSP method is used to detect aberrant promoter hypermethylation of p16. Negative and positive controls for methylated and unmethylated reactions were performed for each set of PCR reactions with plasma DNA. |  |
| Leung 2005 | Forty-nine patients (18 male; mean age 57 yr, range 47–80yr) who were newly diagnosed to have sporadic cancer of the colon or rectum were included with tumor stage TNMⅠ-Ⅳ | serum | All blood was drawn at the time of diagnosis before any therapeutic intervention. | Serum was immediately separated and stored at −80◦C prior to processing. | Genomic DNA  was extracted from 800 µL serum with a commercially available DNA extraction kit. | Three tumor-related genes  were examined by MethyLight. |  |  | CpGenomeTM Universal methylated DNA (Chemicon International Inc., CA, USA) was included as positive  control in all amplifications and internal reference whereas | A standard curve was created by plotting the logarithmic amount of the standard universal methylated DNA  (31.25 pg to 10 ng) against the threshold cycle (CT) value.  The minimal correlation coefficient was 0.98 in all amplifications. The corresponding amount of methylated DNA per  µL of the serum samples was determined from the standard  curve.  Determination of  cutoff values for each serum methylation marker was made by  the receiver-operator characteristics (ROC) curve. |
| Messaoudi 2016 | metastatic colorectal cancer (mCRC) patients | plasma | 4 mL blood samples were collected in K3 EDTA tubes. Plasma was isolated within one hour after drawing blood. The isolation process consisted in a two-step centrifugation. First, blood tubes were centrifuged for 10 min in a Heraeus Multifuge LR centrifuge with a speed spin of 1200 g and a temperature of 4°C. Supernatant was collected, and the buffy coat was avoided with precaution. The collected supernatant was centrifuged a second time to remove any possible remaining cells. This second centrifugation step was performed for 10 min at 4°C and with a spin speed of 16,000 g. | Plasma supernatant was then transferred in a 1.5 mL tube, extracted immediately after or stored at -20°C | CcfDNA extraction was performed with the QIAGEN blood mini kit, and by following the main steps of the “Blood and body fluid protocol”. However, we modified two elements: during the extraction, 1 mL of plasma was processed sequentially in one column. Then, ccfDNA was eluted in 130 μL of elution buffer. Freeze-thawing was avoided to reduce fragmentation of the eluted ccfDNA, and no extracts were conserved for more than three months at -20°C. | Intplex |  | DNA from the cells harboring targeted mutation was serially diluted six times in high-concentrated WT genomic DNA from human placenta (Sigma Aldrich) up to a dilution of 2 mutated copies in 20,000 WT copies. | Non-template controls were performed in each experiment for the different primer sets. Positive controls for mutation assessment were also added to each PCR run. These controls are genomic DNA from cell lines with known mutations. The respective correspondence between cell lines and the corresponding mutation was further detailed: HCT-116 for the G13D KRAS mutation, SW620 for the G12V KRAS mutation, A549 for the G12S KRAS mutation, LS174T for the G12D KRAS mutation, MiaPaca2 for the G12C mutation, SW1116 for the G12A KRAS mutation, and HT29 for the V600E BRAF mutation. Synthetic DNA bearing the KRAS sequence of interest (Horizon Discovery Ltd) was used as a positive control for KRAS G12R. |  |
| Philipp 2012 | CRC stage I-Ⅳ | Serum | Serum samples from 311 patients with CRC drawn before initiation of therapy. | Blood samples  were obtained pre-therapeutically and were transported by a  shock absorbed tube mailing system within 15–30 min after  blood drawing to the central laboratory. All specimens were  centrifuged at 2,000g at 4 C for 10 min. The supernatant was  transferred into polypropylene cryotubes and stored frozen at  −80 ◦C. | The frozen serum samples were thawed at room temperature  and homogenized by smoothly flipping the tube containing the serum. Genomic DNA from 200 ll of each serum sample  was isolated using the High Pure Viral Nucleic Acid Kit  (Roche Applied Science, Mannheim, Germany) according to  the manufacturer’s instructions and eluted in 50 ul of Elution  Buffer. | Bisulfite-treated DNA was analyzed by a fluorescence-based, real-time PCR assay, described previously as MethyLight | The specificity of all  reactions for methylated DNA was confirmed by separately  amplifying completely methylated and unmethylated human control DNA (Chemicon, Temecula, CA) |  | Dispersed Alu repeats were used to control for  DNA amplification and to normalize for input DNA | The percentage of fully methylated reference (PMR) at a specific locus was calculated as described  previously 29 by dividing the gene/Alu ratio of a sample by the gene/Alu ratio of fully methylated, bisulfite-treated DNA  (CpGenomeTM Universal Methylated DNA, Millipore, Billerica, MA) and multiplying by 100. A gene was considered  methylated if the percentage of the fully methylated reference  value was >0 |
| Sefrioui 2015 | 34 consecutive patients undergoing a chemotherapy  regimen for MCRC | plasma | Samples were systematically collected immediately before a chemotherapy course and the timing during the  treatment sequence was not standardized.All patients, except two,  received at least one course of chemotherapy before sample collection with a median of 4 courses (range 0–16) | Blood samples were processed in the two hours following blood  collection. Plasma was obtained after two successive centrifugations of peripheral blood samples at 2700 × g for 20 min at room. temperature and then at 16,000 × g for 10 min at 4 ◦C; the samples were then stored at −80 ◦C until DNA extraction. | DNA was  extracted from 1 to 3 mL of plasma using the QIAamp® Circulating  Nucleic Acid kit (Qiagen, Hilden, Germany) according to the manufacturer’s instructions, eluted in a final volume of 30 l and stored  at −20 ◦C. | ctDNA measurements by dPCR were performed using  the QuantStudioTM 3D Digital PCR System. For experiments with patient  samples, all dPCR assays were conducted in duplicate by two independent operators except for 4 patients because of insufficient  material. |  |  | Real-time quantitative PCR  (qPCR) was performed using a competitive allele-specific TaqMan®  PCR assay (castPCRTM Technology, Life Technologies, Carlsbad, CA,  USA), which is based on real-time quantitative clamp-based PCR  technology comprising a gene reference assay and a mutant allele  assay |  |
| Spindler 2013 | histopathologically  verified metastatic colorectal cancer was included, with  age >18 years. Patients with other  concurrent cancer diseases (within 5 years of inclusion, apart  from squamous cell carcinoma of the skin), having received  experimental therapy within 30 days prior to inclusion, or with planned  radiotherapy to target lesions were not eligible. | serum | pretreatment blood samples were drawn  priorw to the first cycle of therapy and at each visit until the time of  progression. | Plasma was obtained from blood samples collected in  EDTA tubes and centrifuged at 2000g for 10 min within 2 h of  collection. Plasma was stored at −80 ◦C until use | DNA was purified from 1 ml of plasma  using a QIAsymphony virus/bacteria midi-kit on a QIAsymphony  robot (Qiagen), according to the manufacturer’s instructions. DNA  was eluted in 110 ul | Analyses of plasma DNA were performed with the in-house  assays that are based on the Amplification Refractory Mutation  System-Quantitative PCR (ARMS-qPCR) methodology |  |  |  |  |
| Trevisiol 2006 | Eighty-six patients with colorectal cancer were enrolled. Criteria for exclusion from the study were the following: in situ colorectal carcinomas, emergency surgical procedures for occlusive or perforated colorectal cancer, synchronous colorectal or noncolorectal cancers, familial adenomatous polyposis or hereditary non-polyposis colorectal  cancer, and patient’s refusal to give informed consent to the  study. kras mutation was analysed in dukes's stage D patients . | serum | Peripheral blood (5-10 mL) was sampled before  surgery, allowed to clot at room temperature for 2 hours,  and centrifuged at 1000 g for 10 minutes. | Peripheral blood was allowed to clot at room temperature for 2 hours,  and centrifuged at 1000 g for 10 minutes. Serum aliquots  were snap frozen and stored at -80°C until processed | serum DNA were extracted on QIAamp  spin columns (Qiagen, Hilden, Germany). | KRAS2 codon 12 gene mutations  in serum samples were detected by mutant-enriched PCR  (ME-PCR). All reactions were carried out at least twice. |  |  | DNA from the SW480 (homozygous for the codon  12 KRAS2 valine mutation, GTT) and LoVo (wild type for  codon 12) cell lines was run with each ME-PCR as positive and negative control. The PCR negative control was DNA-free PCR mix. |  |
| Bazan 2006 | patients  undergoing resective surgery for primary operable CRC at a single  institution. Inclusion criteria were:  histologically-confirmed CRC diagnosis; and patients undergoing radical  surgery with resection margins histologically negative for neoplastic  infiltration. | plasma | pre-operative blood sample | The plasma sample was obtained by centrifuging 3 ml of peripheral blood at  1550 g for 30 min. An aliquot was prepared from the resulting supernatant  and was stored at –20 C until required | Genomic DNA was extracted by  means of an Ultrasense Virus Kit (Qiagen, Hilden Germany) | Direct automatic sequencing was used to detect the  same genetic alterations in the plasma.Genomic DNA obtained from plasma was  modified with the CpGenome DNA Modification kit (Intergene Company)  following the manufacturer’s instructions |  |  | Peripheral blood leukocytes  (L) were used as negative controls and universal methylated DNA,  UMD (ONCOR, Geithersburg, MD) was used as a positive control |  |
| Herbst 2009 | colorectal cancer stages  International Union Against Cancer (UICC) I, II and  III | serum | In each case serum was drawn before surgery. | Blood was centrifuged at 3000 g for 10 min at room temperature. Aliquots of blood were stored at – 80°C |  | Bisulfite treated DNA was analysed by a fluorescence-based,  real-time PCR assay, described earlier as MethyLight. | Specificity  of the reactions for methylated DNA was confirmed by  separately amplifying completely methylated and unmethylated human control DNA |  | The reference gene, beta-actin (ACTB), was used to control for DNA  amplification and normalize for input DNA | The percentage of methylated reference at a specific locus was calculated as described earlier [23] by dividing the gene: actin ratio of a sample by the gene: actin ratio of fully methylated DNA and multiplying by 100. A gene was deemed methylated if the percentage of the fully methylated reference value was greater than 0 |
| Tie 2014 | Stage II CC patients | plasma | plasma samples are being collected at 4-10 weeks post-op in 250 stage II CC patients, with serial 3 monthly samples on a subset of 175 patients |  |  | massively parallel sequencing platform (Safe-SeqS) |  |  |  |  |
| Lee 2013 | CRC stage Ⅰ-Ⅳ | plasma | The plasma specimens  were collected from the CRC patients before surgical treatment, chemotherapy, or radiation therapy. Blood samples were collected from remnants of routine blood samples of two 9-ml K2-EDTA bottles. The specimens were processed and  stored within 24 hours of collection. Whole-blood specimens were  kept at 2 to 8°C before centrifugation and held at 15 to 30°C for  approximately 30 minutes immediately before centrifugation. | The  blood samples were centrifuged in the blood tubes at room temperature for 10 minutes at 1500g and recentrifuged at 1500g for 10 minutes in 15-ml tubes. The buffy coat layer was not to be disturbed while  transferring the plasma. The plasma specimens were stored at −70°C  before the analysis. At least 4 ml of plasma was collected for testing. | DNA was extracted by using the Abbott m2000sp Sample Preparation System (Abbott Molecular, Abbott Park, IL).The patient specimens and one each of the positive and negative controls included  in the Abbott RealTime mS9 Colorectal Cancer Assay Kit (Abbott  Molecular) were processed with the Abbott m2000sp. | Bisulfite modification was performed by Abbott Bisulfite Modification Kit according to the manufacturer’s protocol. |  |  | The positive and negative controls provided by the manufacturer were processed along with the samples from the DNA extraction stage. | Samples were reported as positive when the internal controls were positive and when at least one of three samples was positive. |
| Ryan 2003 | A total of 123 patients with  a confirmed histological diagnosis of colorectal neoplasia  (ranging from severely dysplastic tubulovillous adenoma  (TVA) to Dukes’ D colorectal cancer) were included in this  study. | Serum | The postoperative blood sampling protocol included a sample taken one week and one month postoperatively, followed  by three monthly samples thereafter until the end of the study  period. | Blood (10 ml) was drawn into serum tubes (Becton-Dickinson  SSAT tubes) and placed immediately on ice. Samples were  kept on ice during all stages of handling. Within two hours,  bloods were centrifuged at 1500 g for 30 minutes at 4°C. Serum  was removed and stored at −20°C until further use. | Qiagen  mini-kit blood DNA extraction kits were used to extract DNA  from sera. | Mutation at codon 12 was analysed using a combination of a  previously described semi nested mutant enrichment  technique and direct sequencing |  |  | PCR  with 5 µl of serum DNA was performed to confirm the  presence of amplifiable quantities of DNA. In all  reactions, two no template controls (sterile water), one  wild-type DNA control (healthy control whole blood DNA),  and one mutant positive control (DNA from SW 480 cell line)  were included. |  |
| Tham 2014 | This prospective study included 150 consecutive patients  with sporadic stage I-III CRCs . Patients with inflammatory bowel disease,  recurrent colorectal cancer, family history suggestive of  Lynch Syndrome defined by Amsterdam criteria, or familial adenomatous polyposis were excluded. | serum | Blood samples are collected longitudinally at 3 time  points: 1 week before surgery, 6-month follow-up (6MFU), and 1-year follow-up (1Y-FU). |  |  | Genomic DNA underwent bisulfite conversion by using  Epi-Tech kit according to manufacturer’s protocol (Qiagen, Hilden, Germany). Bisulfite-converted DNA was  subjected to subsequent methylation-specific quantitative  PCR (qPCR) as described. Each  reaction was run in duplicate or triplicate. |  | Assay sensitivity was  defined as the proportion of recurrent cases that had serum levels above cutoff values. Sensitivity differences were  examined by McNemar’s test. Association between methylation levels in sera and tumors was evaluated using  Spearman’s correlation test. | Every plate  included a positive control and a no-template control. Methylation levels of genes of interest were normalized by  dividing the gene/ACTB ratio of a sample by the gene/  ACTB ratio of a positive control and multiplying by  1000. Quantities of 7 target genes  and 1 control gene b-actin (ACTB) were interpolated  from respective standard curves constructed from 5 to 6  serial dilutions of a methylated DNA standard. | ROC curves  for recurrence detection were constructed and optimal  cutoff values of serum methylation markers were determined based on Youden index. |
| Wallner 2006 | The gene evaluation set consisted of  38 patients with newly diagnosed sporadic colorectal cancer, of which 24 were M0 (age range, 34-87 years; mean, 67 years) and 14 were M1  (age range, 33-72 years; mean, 62 years), | serum | In each case, serum was  drawn before any therapeutic intervention | The blood was centrifuged  at 3000 g for 10 min at room temperature. Aliquots of serum were stored  at -80°C | Genomic DNA from 1 mL of  each serum sample was isolated using the QIAamp DNA Blood mini kit  (Qiagen, Hilden, Germany) according to the manufacturer’s instructions. | Bisulfite-treated DNA was analyzed by  a fluorescence-based, real-time PCR assay, described previously as  MethyLight | Specificity of the reactions  for methylated DNA was confirmed by separately amplifying completely methylated and unmethylated human control DNA (Chemicon,  Temecula, CA) with each set of primers and probes |  | The reference gene, b-actin (ACTB),was used to control for DNA  amplification and normalize for input DNA | The percentage of methylated reference at a specific locus was calculated as described previously  by dividing the gene/actin ratio of a sample by the gene/actin ratio of fully methylated DNA and multiplying by 100. A gene was deemed methylated if the percentage of the fully methylated reference value was >0. |
| Matthaios 2016 | Patients were suffering from either early oper- able (88/155, 56.8%) or metastatic disease (67/155, 43.2%). | Serum | Sample collection and isolation of cell-free DNA. Whole blood was extracted from patients pre-operatively. | Blood was collected in serum clot activator tubes. Serum was obtained immediately through centrifugation at 3,000 x g for 10 min and stored at -80 ̊C until DNA extraction. | Cell-free DNA from serum samples was isolated using the High Pure Viral Nucleic Acid kit (Roche Diagnostics GmbH, Mannheim, Germany). A total of 300 μl serum were mixed with 300 μl working solution and 60 μl proteinase K (18 mg/ml), and incubated for 10 min at 72 ̊C; DNA isolation was then processed as described in the manufacturer's protocol. DNA concentration was determined with an ND-100 spectrophotometer (NanoDrop Technologies; Thermo Fisher Scienti c, Inc., Wilmington, DE, USA). | Sodium bisul te conversion of ≤200 ng cell-free DNA was performed using the EZ DNA Methylation-GoldTM kit (Zymo Research Corporation, Irvine, CA, USA), according to the manufacturer's protocol. The converted DNA was stored at -80 ̊C until used. The methylation status of APC and RASSF1A in cell-free circu- lating serum DNA samples was detected by MSP using speci c primer pairs for both the methylated and unmethylated promoter sequences. Each MSP reaction was performed in a total volume of 25 μl. Sodium bisul te‐converted DNA (1 μl) was added into a 24-μl reaction mixture that contained 0.1 μl Taq DNA polymerase (5 U/μl; GoTaq® Hot Start Polymerase; Promega Corporation, Madison, WI, USA), 5 μl 10X buffer, 2.0 μl MgCl2 (50 mmol/l), 0.5 μl deoxynucleotides triphos- phate (10 mmol/l; Fermentas; Thermo Fisher Scienti c, Inc., Pittsburgh, PA, USA) and 1 μl each of the corresponding forward and reverse primers (10 μmol/l); lastly, distilled H2O was added to a nal volume of 25 μl. Sodium bisul te‐treated DNA was ampli ed in two separate MSP reactions, one with a set of primers specific for methylated DNA, and one for unmethylated promoter sequences. |  |  | Human placental genomic DNA (gDNA; Sigma-Aldrich, St. Louis, MO, USA) methylated in vitro with M.SssI methylase (New England BioLabs, Inc., Ipswich, MA, USA) was used, following sodium bisul te conversion, as a fully methylated (100%) MSP positive control. The same unmethylated placental gDNA was used, following sodium bisul te conversion, as a negative MSP control. | MSP products for methylated and unmethyl- ated promoters were fractionated on 2% agarose gels containing 40 mM Tris-acetate/1.0 mM ethylenediaminetet- raacetic acid (pH 8.0) and visualized by ethidium bromide staining. |
| Liu 2016 | This prospective study included 165 consecu- tive patients with sporadic CRCs who under- went elective curative surgical resection in a single institution (Singapore General Hospital) between Oct 2003 and June 2005. Patients who underwent neoadjuvant chemotherapy or radiotherapy, with in ammatory bowel diseas- es or family history suggestive of Lynch syn- drome or familial adenomatous polyposis were excluded. | Serum | Peripheral blood was obtained within one week before surgery. | Serum was separated from the clot by centrifugation (at 1832 g for 12 min) within 30–45 min of blood collection.  Aliquoted serum was stored at 80 C until DNA isolation by a sodium iodide-based protocol described previously (Fong et al., 2009). | DNA isolation by a sodium iodide-based protocol | DNA was converted by bisulfate and subjected to uorescence-based quantitative PCR (qPCR) as previously described |  |  | qPCR on seven target genes and one control gene b-actin (ACTB) was carried out in a 7500 Sequencing Detection System (Applied Biosystems, Foster, CA). Each reaction was run in duplicates or trip- licates. Every plate also included a positive con- trol and a no template control. | Quantities of genes of interest were normalized by dividing the gene/ACTB ratio of a sample by the gene/ ACTB ratio of a positive control and multiplying by 1000. |
| Lin 2016 | Initially, clinical data and tissue samples of 568 CRC patients who underwent surgery between 2008 and 2009 were obtained from the Biobank of the Taipei Veterans General Hospital, a prospectively established biobank. After excluding patients who underwent preoperative chemoradiotherapy (n = 45), neoadjuvant chemotherapy (n = 36), emergent operative procedures (n = 32), or evidence of familial adenomatous polyposis (n = 4), 451 CRC patients were enrolled in this study. | plasma | Plasma was collected before surgery | plasma was immediately frozen and stored in liquid nitrogen | cfDNA from 1 mL plasma from each case was extracted using the QIAamp Minelute Virus Kit (Qiagen) according to the manufacturer’s recommendations; DNA quantity was confirmed using PicoGreen (Life Technolo- gies, Carlsbad, CA, USA) and a VICTOR3V Spectrophotometer (PerkinElmer, Inc., Waltham, MA, USA). | After bisulfite conversion (EpiTect Fast 96 DNA Bisulfite Kit, Qiagen), whole-genome amplification, enzymatic frag- mentation, precipitation, resuspension, and hybridization, the intensity data were acquired with an Illumina HiScan scanner. The image was processed using the GenomeStu- dio Methylation module (Illumina) to obtain the b value of each CpG site. |  |  |  |  |
| Tie 2016 | Eligible patients had a staging CT chest/abdomen/ pelvis within 12 weeks of study entry to exclude metastatic disease. Patients with a previous malignancy within the last 5 years were excluded. | plasma | Blood samples for ctDNA analysis were collected at 4 to 10 weeks postoperatively, with serial three-monthly blood samples collected for up to 2 years from a subset of patients. |  | Plasma (10 ml) was purified from each patient using the QIAamp Circulating Nucleic Acid kit (Qiagen cat. no. 55114). | To distinguish genuine mutations in the samples from artifactual variants arising from sequencing and sample preparation steps, we used Safe-SeqS, an error reduction technology for detection of low-frequency mutations (33). In the Safe-SeqS assay, plasma DNA was aliquoted into 24 wells of a 96-well plate so that an average of 0.5 to 3 ng of DNA was contained in each well. The DNA from each well was then amplified (15 cycles) using primers containing unique identifier sequences (UIDs), which consisted of 14 random bases with an equal probability of A, C, T, and G, to allow for the distinction of each template molecule. The am- plified reactions were purified with AMPure XP beads (Beckman Coulter) and eluted in 250 ml of Buffer EB (Qiagen). One percent (2.5 ml) of purified PCR product was then amplified in a second round of PCR with universal primers, as previously described. The PCR products were purified with AMPure and sequenced on an Illumina MiSeq instrument. | DNA from the peripheral blood lymphocytes of healthy individuals was used as a control in each experiment to identify potential false-positive mutations. |  |  | The one-sided test was used to avoid attributing significance to a ctDNA-negative sample that has fewer supermutants than the asso- ciated control. A 0.1 P value was then chosen as the threshold to classify a sample of interest as ctDNA-positive (P < 0.1) or ctDNA-negative. Given the lack of a gold standard, a specificity of at least 0.90 was considered desirable, and a P value equal to 0.1 yielded 0.90 specificity when performing leave-one-out cross-validation on the controls. |
| Wang 2004 | 104 nonselected patients who underwent surgical re- section for CRC | Serum | 5 ml samples of peripheral blood were obtained from the 104 CRC pa- tients at the time of surgical resection | Serum was separated and stored at −20°C before the DNA extraction | DNA from serum samples was purified using the Puregene kit (Gentra Systems, Minneapolis, MN, USA) according to the manu- facturer’s protocol | The PCR products were purified by the QIAEX II Gel Extraction Kit (Qiagen, Valencia, CA, USA) and then subjected to sequencing using a double-stranded cycle sequencing system (GIBCO, Gai- thersburg, MD, USA). The purified products were then sequenced directly with a T7 promoter/IRD800 (LI-COR, Lincoln, NE, USA), which is a T7 promoter primer labeled with a heptamethine cyanine dye, or using DNA polymerase incorporating IRD-labeled dATP for the sequencing reaction. Upon completion of the se- quencing reaction, 4 μl of formamide loading buffer was added to the reaction mixture; the sample was heated to 95°C for 5 minutes, snap-cooled, and loaded onto the sequencing gel.  An automated DNA electrophoresis system (model 4200; LI-COR) with a laser diode emitting at 785 nm and fluorescence detection between 815 and 835 nm was used to detect and analyze the sequencing ladder. |  |  | DNA from 50 healthy volunteers was used as negative control |  |
| Lindforss (2005) | Twenty-five patients with CRC, age 49-90 years (median age 72 years), undergoing surgery at our department, were studied | plasma | Blood samples were taken preoperatively and 3 days after surgical resection | Plasma was separated after centrifugation at 3000 rpm immediately after sampling and stored at -70ÆC until further use. | The samples were then condensated in microtubes, and genomic DNA was extracted with the specific Qiamp DNA Blood kit (Qiagen), according to the manufacturer. | TGGE of the purified amplification products was performed in a system (20) patented by Qiagen and produced by Biometra (Göttingen, Germany). We used horizontal 8% gels with 4.2 g urea, 2.0 ml acrylamide (stock solution 37.5:1, 40%), 0.1 ml TBE (conc. x 10), 0.5 ml glycerol (50%), 22.5 Ìl TEMED and 42 ml APS (4%) in distilled water, filled up to 10 Ìl. Prior to screening of the amplified k-ras samples, perpendicular TGGE made it possible to identify the different alleles by their individual melting behavior directing at which temperature the different DNA strands would separate. The screening of the multiple samples was done on plates with pre-fixed slots for eight and twelve samples using parallel TGGE. |  |  | DNA extracted from SW480 cells, expressing k- ras mutations in codon 12 and 13 (21), was used as positive control. |  |
| Lin(2014) | Our study enrolled 191 colorectal cancer patients who received surgery at Taipei Veterans General Hospital from 2005 to 2008 | plasma | Plasma was collected before surgery | Plasma was immediately frozen in liquid nitrogen and stored in Biobank of Taipei Veterans General Hospital | DNA from plasma was extracted using the QIAamp DNA Tissue Kit and Minelute Virus Kit (Qiagen, Valencia, CA), respectively, according to the manufacturer’s recom- mendations. The quality and quantity of DNA were confirmed using the Nanodrop 1000 Spectrophotometer (Thermo Scientific) | The MassDetect CRC panel (v1.0), enabling the identification of 155 mutations in 74 genes (Supple- mentary Table 1), was designed according to previous study.11 PCR and extension primers for the mutations were designed using the MassArray Assay Design 3.1 software (Sequenom, San Diego, CA). The mutation alleles were manually designed by extension in either the forward or reverse direction to have lower mass than the reference allele. After analyzing the primer designs with BLAST, any necessary modification was made in order to avoid pseudogene amplification. The details of MassARRAY-based mutation detection methods are described in Supplementary Table 1. The PCR products of multiplexed reactions were spotted onto SpectroCHIP II arrays, and DNA fragments were resolved by on the MassARRAY Analyzer 4 System (Sequenom). Each spectrum was then analyzed using the Typer 4.0 software (Sequenom) to call mutations. Putative mutations were further filtered by manual review |  |  |  |  |
